# Differences in Home Health Services and Outcomes Between Traditional Medicare and Medicare Advantage

**DOI:** 10.1001/jamahealthforum.2023.5454

**Published:** 2024-03-01

**Authors:** Rachel A. Prusynski, Anthony D’Alonzo, Michael P. Johnson, Tracy M. Mroz, Natalie E. Leland

**Affiliations:** 1 Department of Rehabilitation Medicine, University of Washington, Seattle; 2 BAYADA Home Health Care, Moorestown, New Jersey; 3 Department of Occupational Therapy, School of Health and Rehabilitation Sciences, University of Pittsburgh, Pittsburgh, Pennsylvania

## Abstract

**Importance:**

Private Medicare Advantage (MA) plans recently surpassed traditional Medicare (TM) in enrollment. However, MA plans are facing scrutiny for burdensome prior authorization and potential rationing of care, including home health. MA beneficiaries are less likely to receive home health, but recent evidence on differences in service intensity and outcomes among home health patients is lacking.

**Objective:**

To examine differences in home health service intensity and patient outcomes between MA and TM.

**Design, Setting, and Participants:**

This cross-sectional study was conducted from January 2019 to December 2022 in 102 home health locations in 19 states and included 178 195 TM and 107 102 MA patients 65 years or older with 2 or fewer 60-day home health episodes. It included a secondary analysis of standardized assessment and visit data. Inverse probability of treatment weighting regression compared service intensity and patient outcomes between MA and TM episodes, accounting for differences in demographic characteristics, medical complexity, functional and cognitive impairments, social environment, caregiver support, and local community factors. Models included office location, year, and reimbursement policy fixed effects. Data were analyzed between September 2023 and July 2024

**Exposure:**

TM vs MA plan.

**Main Outcomes and Measures:**

Home health length of stay and number of visits from nursing, physical, occupational, and speech therapy, social work, and home health aides. Patient outcomes included improvement in self-care and mobility function, discharge to the community, and transfer to an inpatient facility during home health.

**Results:**

Of 285 297 total patients, 180 283 (63.2%) were female; 586 (0.2%) were American Indian/Alaska Native, 8957 (3.1%) Asian, 28 694 (10.1%) Black, 7406 (2.6%) Hispanic, 1959 (0.7%) Native Hawaiian/Pacific Islander, 237 017 (83.1%) non-Hispanic White, and 678 (0.2%) multiracial individuals. MA patients had shorter home health length of stay by 1.62 days (95% CI, −1.82 to 1.42) and received fewer visits from all disciplines except social work. There were no differences in inpatient transfers. MA patients had 3% and 4% lower adjusted odds of improving in mobility and self-care, respectively (mobility odds ratio [OR], 0.97; 95% CI, 0.94-0.99; self-care OR, 0.96; 95% CI, 0.92-0.99). MA patients were 5% more likely to discharge to the community compared with TM (OR, 1.05; 95% CI, 1.01-1.08).

**Conclusions and Relevance:**

The results of this cross-sectional study suggest that MA patients receive shorter and less intensive home health care vs TM patients with similar needs. Differences may be due to the administrative burden and cost-limiting incentives of MA plans. MA patients experienced slightly worse functional outcomes but were more likely to discharge to the community, which may have negative implications for MA patients, including reduced functional independence or increased caregiver burden.

## Key Points

**Question:**

Are there differences in service intensity and outcomes of home health care between Medicare Advantage and traditional Medicare?

**Findings:**

In this cross-sectional study of 178 195 traditional Medicare and 107 102 Medicare Advantage patients, Medicare Advantage patients had shorter home health lengths of stay and fewer nursing, therapy, and aide visits compared with similar patients with traditional Medicare. Medicare Advantage patients were more likely to be discharged to the community compared with Traditional Medicare but had lower likelihoods of improving in self-care and mobility function.

**Meaning:**

The results of this study suggest that Medicare Advantage patients receive fewer home health visits and have worse functional outcomes compared with traditional Medicare.

## Teaser

This cross-sectional study examines differences in home health service intensity and patient outcomes between Medicare Advantage and traditional Medicare.

## Introduction

The Medicare home health care benefit is provided in the home environment to homebound individuals in need of care for an illness or injury.^1^ This benefit includes nursing, rehabilitation, home health aides, and medical social work services. Home health care can be provided after a preceding hospitalization or without hospital admission if care needs are identified by a clinician. Home health may substitute for care in institutions such as skilled nursing or inpatient rehabilitation facilities.^2,3^ Compared with no home health, receiving home health has been associated with positive outcomes, including lower mortality, reduced hospital readmissions, and functional improvement.^4,5,6,7^ As part of the Medicare benefit, home health is provided to more than 3 million traditional Medicare (TM) beneficiaries annually, at a cost of nearly $17 billion in 2021.^1^

Private insurers are increasingly administering Medicare benefits, including home health, through Medicare Advantage (MA) plans.^8^ Enrollment in MA recently surpassed TM, with 51% of the eligible Medicare population enrolled in an MA plan in 2023.^9^ Medicare pays MA plans a capitated rate per beneficiary to cover enrollee health needs, thus incentivizing MA plans to coordinate care and reduce costs.^10,11^ Benefits vary substantially across the nearly 4000 different MA plans offered in the US, but all plans must meet certain minimum standards, which include home health coverage. MA plans may also provide benefits, such as vision and dental, that TM does not cover.^10,12^ However, MA plans are known to reduce costs by requiring copays, limiting networks of providers, and requiring prior authorization.^10,13,14,15^

Within the current payment policy for TM, beneficiaries are certified for 60-day home health episodes, and agencies are reimbursed for 30-day payment periods, with rates based on patient characteristics (clinical category, functional status, and comorbidities), period timing (early vs late), and admission source (institution vs community).^16^ Payment is intended to cover all visits from different care clinicians during the 30-day period. In contrast, MA plans have more flexibility in limiting costs by requiring prior authorization or frequent recertifications, constraining the number of visits, or limiting the length of the home health stay.^11^ Compared with TM, MA beneficiaries have less frequent home health utilization and shorter home health stays, even when home health is prescribed at hospital discharge.^11,17,18,19,20^ Concerns about reduced access to care, including home health, within MA motivated a recent rule from the US Centers for Medicare & Medicaid Services requiring MA plans to comply with Medicare coverage requirements, limit prior authorization criteria, and reduce care delays.^21,22^

In addition to lower home health utilization, MA beneficiaries receive care from lower-quality agencies compared with TM enrollees.^11,23^ However, it remains unclear how differences in agency-level quality are associated with individual patient outcomes. Previous studies examining differences in home health outcomes comparing TM and MA have been mixed. Compared with TM enrollees, one 2015 to 2016 study found lower rehospitalization rates for MA patients with heart failure, stroke, and joint replacement, but higher mortality rates for MA patients with stroke and joint replacement.^11^ A similar study found lower rehospitalization rates for all diagnoses after home health within MA compared with TM.^11^ Another study of 1 MA plan found positive home health outcomes for MA compared with TM, with lower rehospitalization, more days at home, and lower costs.^24^ One study found lower reports of functional improvement within MA compared with TM, but relied on a small survey sample that pooled skilled nursing and home health users together.^25^ There is a need to further examine discharge to the community (vs an institution) and improvements in self-care and mobility, which are essential targets of rehabilitation services in home health.^1^

Previous work has also not adequately accounted for substantial differences between TM and MA patient populations. It is well established that MA plans attract healthier beneficiaries compared with TM.^26,27^ While studies use various methods to adjust for differences in disease severity, to our knowledge, few account for differences in social supports or cognitive and functional characteristics that are associated with home health outcomes.^11,24^ Finally, higher levels of marginalized beneficiaries, including patients of Black and Hispanic racial and ethnic groups, are enrolling in MA compared with TM.^28,29,30^ These groups experience worse clinical care compared with non-Hispanic White patients and worse access to quality home health care,^30,31,32^ which is associated with several factors, including rurality and community deprivation.^31,32,33,34,35,36^ Thus, potential disparities in home health services and outcomes between TM and MA must be considered within the local community context of each beneficiary.

This study addresses these gaps by using administrative data from a large national health care network to examine differences in home health care intensity (ie, length of stay, number of visits) and patient outcomes between patients with MA and TM after admission to home health. We examine self-care and mobility function, inpatient readmissions during the home health stay, and community discharge while accounting for a comprehensive set of demographic, clinical, and community factors.

## Methods

### Data Sources and Sample

Deidentified data were acquired through a data use agreement with a large national nonprofit home health company with 102 locations operating in 19 states. Thus, this study was exempted by the University of Washington institutional review board, and informed consent was waived. The company provided data for all home health patients with TM or MA plans who had complete admission and discharge assessments from January 2019 through December 2022. Data included the Outcomes and Assessment Information Set (OASIS) and information on number of visits by discipline and occurrence of inpatient transfers. The study followed the Strengthening the Reporting of Observational Studies in Epidemiology (STROBE) reporting guidelines.

We limited the sample to patients 65 years or older with complete data who received 2 or fewer 60-day certification episodes. This comprised most of the patients and allowed us to exclude patients seen for intermittent maintenance care. For patients who were recertified for a second 60-day episode, we used the admission OASIS from the first episode and the discharge assessment from the final episode to capture their entire stay.

### Outcomes

Home health intensity outcomes included length of stay (LOS), defined as the number of days between admission and discharge from home health, and number of visits from the following disciplines: nursing, physical therapy, occupational therapy, speech therapy, social work, and home health aides. Patient outcomes included dichotomous indicators for discharge to the community (vs an institution), transfer to an inpatient facility during the home health stay, and functional improvement on OASIS self-care and mobility scores. Self-care and mobility scores were summed at admission and discharge using 9 OASIS items (M1800-M1870) that rated patients’ independence with self-care tasks, including grooming, upper and lower body dressing, bathing, feeding, and toileting hygiene, and mobility tasks, including transfers from bed to chair, toilet transfers, and locomotion/ambulation. Total self-care scores ranged from 0 to 23, and mobility scores ranged from 0 to 15, with higher scores indicating more impairment.^37^ For each measure, functional improvement occurred if a patient had less impairment on the total score at discharge compared with admission.

### Covariates

We used admission OASIS assessments for all patient characteristics. Demographic characteristics included age, sex (female or male) and self-reported race and ethnicity (American Indian/Alaska Native, Asian, Black, Hispanic, Native Hawaiian/Pacific Islander, non-Hispanic White, or multiracial). We included an indicator for whether patients were admitted after an inpatient stay vs from the community.^38^

We included total self-care and mobility scores at admission and level of cognitive impairment, which was categorized into none, mild, or moderate to severe.^34,37,39^ We included indicators for cognitive or behavioral symptoms at least once per week (ie, memory deficits, impaired decision-making, verbal disruption, physical aggression, or disruptive behavior), pain that interfered with activity, and 2 or more falls or a fall resulting in injury during the previous 12 months.^37^

To reflect medical complexity and health care use, we calculated a weighted Elixhauser Comorbidity Index score from active medical diagnoses in the admission OASIS.^40^ We included indicators for whether the patient had 2 or more hospitalizations during the previous 6 months, was taking 5 or more medications, or had a pressure ulcer or surgical wound at home health admission. Level of dyspnea was categorized as not short of breath, short of breath when walking more than 20 feet or climbing stairs, short of breath with moderate exertion, short of breath with minimal exertion, or short of breath at rest. Incontinence level was categorized as not incontinent, incontinent, or required a urinary catheter.

We used OASIS information on patients’ home environment and level of support, including an indicator for whether home health was provided in a community setting vs an institution (ie, long-term care), whether the patient lived alone, and the availability of assistance at home, which was categorized as none, some, or around the clock. Finally, we included community characteristics that could be associated with home health intensity and/or patient outcomes.^31,34,35,36^ We used rural-urban commuting area codes to categorize patient zip codes as urban, large rural, small rural, or isolated rural.^41^ We included the Social Deprivation Index for the patients’ zip code tabulation area.^42^ Social Deprivation Index scores ranged from 0 to 100, with higher scores indicating higher levels of local socioeconomic disadvantage across categories such as poverty, employment, housing, and access to transportation as derived from the American Community Survey.

### Analysis

Consistent with previous methods that examined differences in postacute care utilization and outcomes between TM and MA, we used inverse probability of treatment weighting (IPTW) to account for differences between groups.^43,44^ Treatment weights were calculated to reflect the propensity of being in the TM or MA group based on all patient and community covariates previously described. After calculating weights, we verified that weighted samples were balanced on all variables.^45^ Next, we used multivariable linear regression to estimate differences in continuous variables (eg, LOS, number of visits) and logistic regression to estimate differences in the probability of dichotomous outcomes (eg, functional improvement) between groups. In each model, we included treatment weights, year and office location fixed effects, and an indicator for whether the stay spanned the implementation of the Patient Driven Groupings Model (PDGM), which was implemented in January 2020 and changed reimbursement incentives for TM episodes.^1^ The year and PDGM fixed effects allowed us to account for the course of the COVID-19 pandemic and declines in home health visits that occurred after PDGM.^1,27^ Location fixed effects also accounted for varying geographical effects of the pandemic and potential differences in managing TM vs MA plans by different office locations. Finally, we used robust standard errors to account for multiple episodes for the same patient. Sensitivity analyses using linear probability models instead of logistic regression for dichotomous outcomes were also performed to test whether model results were sensitive to model specifications. Linear probability models used the same treatment weights and fixed effects as logistic models.

Analyses were conducted in RStudio, version 2023.06.0 (R Foundation), with statistical significance at a 2-sided α < .05.

## Results

Descriptive statistics for outcomes and all covariates for the 178 195 TM patients and 107 102 MA patients are detailed in Table 1. Compared with TM, MA patients were younger, more likely to be admitted from the hospital (vs community), and more likely to identify as male and non-Hispanic White. MA patients had lower medical risk, less impairment in self-care and mobility at admission, and lower rates of cognitive impairment, incontinence, dyspnea, pain, pressure ulcers, and other risk factors, including frequent falls, emergency department (ED) visits, and behavioral symptoms. MA patients were more likely to live alone, less likely to have around-the-clock support at home, and were more likely to live in rural and disadvantaged areas.

**Table 1.** Descriptive Statistics for Patient, Home, and Community Characteristics for Beneficiaries Receiving Home Health From a Nonprofit Company Between 2019 and 2022

Characteristic	No. (%)		
	Traditional Medicare (n = 178 195)	Medicare Advantage (n = 107 102)	*P* value^a^
Demographic			
Age, mean (SD), y	79.8 (10.9)	77.8 (11.3)	<.001
Female sex	113 587 (63.7)	66 696 (62.3)	<.001
Male sex	64 608 (36.3)	40 406 (37.7)	<.001
Admitted from inpatient facility	108 144 (60.7)	68 288 (63.8)	<.001
Receiving HH in a community setting (ie, private home or assisted living)	177 727 (99.7)	106 637 (99.6)	<.001
Race and ethnicity			
American Indian/Alaska Native	379 (0.2)	207 (0.2)	<.001
Asian	4600 (2.6)	4357 (4.1)	<.001
Black	13 591 (7.6)	15 103 (14.1)	<.001
Hispanic	3592 (2.0)	3814 (3.6)	<.001
Native Hawaiian/Pacific Islander	798 (0.4)	1161 (1.1)	<.001
Non-Hispanic White	154 899 (86.9)	82 118 (76.7)	<.001
Multiracial	336 (0.2)	342 (0.3)	<.001
Function and clinical			
Admission mobility function score (0-15), mean (SD)^b^	9.0 (2.3)	8.8 (2.3)	<.001
Admission self-care function score (0-23), mean (SD)	14.4 (3.2)	14.1 (3.2)	<.001
Cognitive impairment			
None	79 142 (44.4)	53 291 (49.8)	<.001
Mild	46 589 (26.1)	26 669 (24.9)	
Moderate to severe	52 464 (29.4)	27 142 (25.3)	
Weighted Elixhauser Comorbidity Index score (−19 to 89), mean (SD)	5.0 (5.4)	4.9 (5.4)	.03
Pain that interferes with activity or movement	146 299 (82.1)	86 102 (80.4)	<.001
Pressure ulcer at HH admission	6996 (3.9)	3754 (3.5)	<.001
Surgical wound at HH admission	37 144 (20.8)	23 359 (21.8)	<.001
Dyspnea level			
Not short of breath	13 945 (7.8%	11 560 (10.8)	<.001
Short of breath with walking more than 20 ft or climbing stairs	23 455 (13.2)	13 970 (13.0)	
Short of breath with moderate exertion	45 777 (25.7)	23 593 (22.0)	
Short of breath with minimal exertion	81 254 (45.6)	49 438 (46.2)	
Short of breath at rest	13 764 (7.7)	8541 (8.0)	
Incontinence level			
Not incontinent	79 597 (44.7)	52 386 (48.9)	<.001
Incontinent	93 424 (62.4)	51 787 (48.4)	
Requires a urinary catheter	5174 (2.9)	2929 (2.7)	
History of ≥2 falls or injurious fall in last 12 mo	77 901 (43.7)	45 253 (42.3%)	<.001
History of ≥2 hospitalizations in the past 6 mo	42 281 (23.7)	25 235 (23.6)	.32
History of ≥2 ED visits in the past 6 mo	40 860 (22.9)	24 422 (22.8)	.44
Currently taking ≥5 medications	164 859 (92.5)	98 322 (91.8)	<.001
Cognitive or behavioral symptoms occurring at least once/wk	67 165 (37.7)	35 270 (32.9)	<.001
Home and community environment			
Availability of assistance at home			
None	2870 (1.6)	2098 (2.0)	<.001
Some	39 865 (22.4)	28 172 (26.3)	
Around the clock	135 460 (76.0)	76 832 (71.7)	
Lives alone	35 158 (19.7)	24 254 (22.6)	<.001
Social Deprivation Index score (1-100), mean (SD)	36.4 (25.6)	43.0 (27.3)	<.001
Rurality			
Urban	165 706 (93.0)	98 808 (92.3)	<.001
Large rural	8432 (4.7)	6256 (5.8)	
Small rural	2357 (1.3)	1259 (1.2)	
Isolated rural	1700 (1.0)	779 (0.7)	

Abbreviations: ED, emergency department; HH, home health.

^a^ *P* values for differences in descriptive statistics by payer were based on unpaired *t* tests for continuous outcomes, proportions tests for dichotomous outcomes, and χ^2^ tests for categorical outcomes.

^b^ Higher scores on mobility and self-care scales indicate higher levels of impairment.

Table 2 includes unadjusted outcomes. MA patients had shorter home health LOS and fewer visits from nursing and therapy disciplines. Compared with TM patients, MA patients were more likely to improve in function and discharge to the community and less likely to transfer to an inpatient facility during their home health stay before adjustment.

**Table 2.** Descriptive Statistics for Episode Characteristics and Unadjusted Outcomes for Beneficiaries Receiving Home Health From a Nonprofit Company Between 2019 and 2022

Characteristic	No. (%)		
	Traditional Medicare (n = 178 195)	Medicare Advantage (n = 107 102)	*P* value^a^
Episode			
Approved for two 60-d periods	21 463 (12.0)	10 961 (10.2)	<.001
Year			
2019	46 513 (26.1)	24 547 (22.9)	<.001
2020	40 759 (22.9)	22 368 (20.9)	
2021	46 890 (26.3)	29 932 (27.9)	
2022	44 033 (24.7)	30 255 (28.2)	
Episode spanned PDGM implementation	5117 (2.9)	2521 (2.4)	<.001
Utilization outcomes, mean (SD)			
Length of stay, d	46.2 (26.1)	43.2 (24.7)	<.001
Nursing visits	4.3 (5.3)	3.8 (4.9)	<.001
Physical therapy visits	7.3 (5.4)	6.8 (4.9)	<.001
Occupational therapy visits	3.1 (3.8)	2.8 (3.5)	<.001
Speech therapy visits	0.6 (2.0)	0.5 (1.8)	<.001
Social work visits	0.2 (0.5)	0.2 (0.6)	<.001
Home health aide visits	0.4 (2.0)	0.4 (1.8)	.08
Patient outcomes			
Improved in mobility function	167 774 (94.2)	101 399 (94.7)	<.001
Improved in self-care function	168 005 (94.3)	101 527 (94.8)	<.001
Discharged to community	165 078 (92.6)	100 411 (93.8)	<.001
Transfer to inpatient facility during episode	13 251 (7.4)	7801 (7.3)	.13

Abbreviation: PDGM, patient-driven groups model.

^a^ *P* values for differences in descriptive statistics by payer are based on unpaired *t* tests for continuous outcomes, proportions tests for dichotomous outcomes, and χ^2^ tests for categorical outcomes.

The IPTW approach was successful in ensuring balanced covariates between the TM and MA samples in adjusted analyses (eAppendix in Supplement 1). Differences in service intensity outcomes after adjustment with IPTW to account for differences between groups are in Table 3. MA patients had a mean home health LOS that was 1.62 days shorter than TM patients (95% CI, −1.82 to −1.42), which equates to a 3.5% shorter LOS compared with the average TM episode. MA patients also received statistically significantly fewer visits from every discipline except for social work. Compared with TM averages, these differences equated to MA patients receiving 4.9% fewer nursing visits, 2.7% fewer physical therapy visits, 2.9% fewer occupational therapy visits, 5.0% fewer speech therapy visits, and 5% fewer home health aide visits.

**Table 3.** Estimated Adjusted Differences in Service Intensity for Home Health MA Beneficiaries Compared With TM Beneficiaries^a^

Service intensity outcome	Estimate (95% CI)	*P* value	TM average	Adjusted MA average	Difference, %
Length of stay, d	−1.62 (−1.82 to −1.42)	<.001	46.2	44.6	−3.5
Nursing visits	−0.21 (−0.25 to −0.17)	<.001	4.3	4.1	−4.9
Physical therapy visits	−0.20 (−0.24 to −0.16)	<.001	7.3	7.1	−2.7
Occupational therapy visits	−0.09 (−0.12 to −0.07)	<.001	3.1	3.01	−2.9
Speech therapy visits	−0.03 (−0.04 to −0.01)	.001	0.6	0.57	−5.0
Social work visits	−0.003 (−0.01 to 0.001)	.12	0.2	0.2	NA
Home health aide visits	−0.02 (−0.03 to −0.002)	.03	0.4	0.38	−5.0

Abbreviations: NA, not applicable; MA, Medicare Advantage; TM, traditional Medicare.

^a^ Linear regression models included inverse probability of treatment weights that included all demographic, clinical, and community characteristics from Table 1 as well as location and year fixed effects, an indicator for whether the home health stay spanned the implementation of the Medicare Patient-Driven Groupings Model in January 2020, and robust standard errors.

Differences in outcomes between groups after adjustment are included in the Figure. Compared with TM, patients with MA were 3% and 4% less likely to improve in mobility function and self-care function, respectively (mobility odds ratio [OR], 0.96; 95% CI, 0.94-0.99; self-care OR, 0.97; 95% CI, 0.92-0.99). MA patients were 5% more likely to be discharged to the community than their TM counterparts (OR, 1.05; 95% CI, 1.01-1.08), and there were no differences in the odds of inpatient transfers between groups. Sensitivity analyses with linear probability models had similar results for all dichotomous outcomes (eAppendix in Supplement 1).

**Figure.**
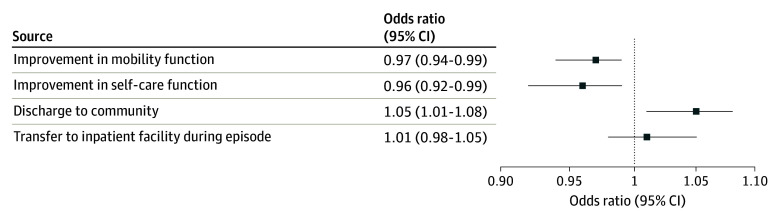
Estimated Adjusted Differences in Outcomes for Home Health Medicare Advantage Beneficiaries Compared With Traditional Medicare Beneficiaries Expressed as odds ratios with 95% CIs. Logistic regression models included inverse probability of treatment weights that included all demographic, clinical, and community characteristics from Table 1, as well as location and year fixed effects, an indicator for whether the home health stay spanned the implementation of the Medicare Patient-Driven Groupings Model in January 2020, and robust standard errors.

## Discussion

In this cross-sectional study of more than 285 000 patients receiving home health from a large, multistate, nonprofit company between 2019 and 2022, we found significant differences in home health service intensity between TM and MA plans after robust adjustment for patient demographic, clinical, and community factors. The differences between groups, in which MA patients were more racially diverse, younger, and significantly less clinically complex, were consistent with prior literature comparing TM and MA populations.^11,43^ The differences in home and community factors between TM and MA, in which MA patients were more likely to live alone, less likely to have assistance, and more likely to live in rural and disadvantaged areas, to our knowledge have not been reported previously for home health. These differences highlight the importance of adequate adjustment across personal and community levels, especially considering the importance of community context and caregiver support for home health outcomes.^31,46,47,48,49^

This study builds on previous research that found lower home health utilization for MA patients compared with TM,^11,17^ which was consistent with strategies to reduce costs of postacute care in MA plans.^25,43^ Building on this past evidence that documented issues with access to home health, the results of the current study go a step further to highlight that, even after admission to home health, MA patients still experienced lower intensity of services compared with TM patients. These results were consistent with other studies that also found shorter home health LOS for MA.^11,17^ Conversely, another study found that MA and TM patients had similar home health LOS and number of visits; however, this study used data from a single MA plan and did not adjust for differences between TM and MA patients for intensity outcomes.^24^ Fewer visits and shorter home health LOS are likely associated with cost-containment strategies used by MA plans, such as cost-sharing or prior authorization requirements for visits beyond a pre-established limit, which do not occur within TM.^11,14,50^

To our knowledge, this is the first study to use standardized assessment data that demonstrates lower adjusted odds of improvement in self-care and mobility function within MA vs TM. Our results are supported by 1 survey study in which MA enrollees reported less functional improvement and lower rates of meeting rehabilitation goals during postacute care compared with TM enrollees.^25^ Consistent with research in other postacute settings,^51,52^ lower functional improvement for MA patients may be associated with fewer therapy visits; however, the direct association between therapy visits and function in home health requires further study. Before adjustment, MA patients had slightly higher rates of functional improvement, but this association reversed after incorporating IPTW. Differences between unadjusted and adjusted functional outcomes reinforce the importance of accounting for a healthier MA population, including incorporating function and a comprehensive set of personal and community factors that are associated with outcomes.^11,53,54,55^

Previous research found lower rehospitalization rates for MA patients compared with TM after home health^11,24,56^; however, we found no difference in inpatient transfers during home health stays. We did not have access to claims data on hospital readmissions after the home health stays ended, so we were not able to replicate analyses of readmissions following discharge from home health. However, our finding of no differences in inpatient transfers during the home health stay for MA and TM may be due to our robust adjustment for clinical complexity and other factors that precipitate inpatient admissions.

In our sample, community discharge rates from home health were higher for MA patients than for TM patients. Prior work found higher rates of community discharge after institutional postacute care for MA patients,^11^ but did not examine community discharge from home health. Higher rates of community discharge are consistent with overall lower utilization of institutional care in MA plans compared with TM.^15,56^ In this study, higher community discharge rates from home health may be cause for concern, as our results demonstrated that MA patients were also more likely to live alone with less support and be discharged with lower odds of functional improvement. This may have negative implications in that MA patients have reduced independence and/or their caregivers have increased burden.^49^

### Limitations

The study data do not account for variability between MA plans, which have different network restrictions, prior authorization requirements, and visit limits.^11^ While we included robust adjustment across demographic, clinical, and community factors to account for selection bias between MA and TM, we did not have access to data from inpatient stays to account for upstream factors associated with medical complexity and cannot account for unobserved differences between these groups. Additionally, issues regarding reliability and validity of outcomes in OASIS assessments have been reported,^57^ including the potential for more incomplete OASIS assessments for MA patients, as OASIS is not used for billing MA plans. However, this risk is mitigated in this study because we used data from 1 home health company that required all OASIS assessments to be complete for all patients regardless of billing requirements for the specific insurance plan. Concerns about OASIS reliability cite different versions of the OASIS that are released every few years; however, we were able to use the same version of the OASIS for all patients in this study. Finally, the self-care and mobility outcomes used in this study are cross-setting measures of function that were implemented in OASIS in 2019 and have been validated in other postacute settings.^37,58,59^ While MA patients were less likely to improve in function compared with TM, the extent to which a dichotomous outcome of functional improvement reflects differences in quality of life or independence is unknown.

Finally, we included data from 102 home health office locations in 19 states; however, all locations are members of 1 nonprofit company, so the results may not be generalizable to independent nonprofit or for-profit agencies, which comprise most of the agencies in the US.^1^ We might expect differences in home health service intensity, and potentially patient outcomes, between TM and MA patients to be even larger in for-profit agencies that are more likely to prioritize cost containment.^60,61,62^ Additionally, using 1 company’s data may further limit generalizability for TM and MA groups, as our sample of 285 000 episodes reflects a small fraction of the millions of home health episodes annually.^1,20^ To our knowledge, no comprehensive recent description of all MA patients in home health has been published, so it is particularly difficult to assess how our sample compares with the entire MA population in home health.^11,63^

## Conclusions

In this cross-sectional study of more than 285 000 patients receiving home health care between 2019 and 2022, compared with TM patients, MA patients had shorter home health LOS, fewer visits by nursing and therapy clinicians and home health aides, lower rates of improving in self-care and mobility function, and higher rates of discharge to the community from home health. Higher rates of community discharge combined with lower functional improvement may have negative associations in terms of independence and caregiver burden for MA patients. Functional outcomes should be included in the evaluation of programs and policies that disincentivize home health utilization, including MA.

## Notes

Supplement 1. eAppendix. Differences in home health services and outcomes between Traditional Medicare and Medicare Advantage

Supplement 2. Data sharing statement
