# Supplementary material for: Differences in Home Health Services and Outcomes Between Traditional Medicare and Medicare Advantage
Source: JAMA Health Forum. 2024 Mar 1;5(3):e235454. doi: 10.1001/jamahealthforum.2023.5454 (PMC10907922; doi:10.1001/jamahealthforum.2023.5454)
Supplement: Supplement 1. — eAppendix. Differences in home health services and outcomes between Traditional Medicare and Medicare Advantage [file jamahealthforum-e235454-s001.pdf]

Supplemental Online Content

Prusynski RA, D’Alonzo A, Johnson MP, Mroz TM, Leland NE. Differences in home health services and outcomes between traditional Medicare and Medicare Advantage. *JAMA Health Forum*. 2024;5(3):e235454. doi:10.1001/jamahealthforum.2023.5454

eAppendix. Differences in home health services and outcomes between Traditional Medicare and Medicare Advantage

This supplemental material has been provided by the authors to give readers additional information about their work.

© 2024 Prusynski RA et al. *JAMA Health Forum.*

eAppendix: Differences in home health services and outcomes between Traditional Medicare and Medicare Advantage

Appendix Table 1. Standardized mean differences (SMD) between Medicare Advantage and Traditional Medicare patients in all covariates used when calculating inverse probability of treatment weights, before and after adjustment

**Demographic Characteristics**	**Unadjusted SMD**	**Adjusted SMD**
Age	-0.1734	0.0015
Female Sex	-0.0304	-0.0015
Admitted from inpatient facility	0.0634	0.0002
Receiving HH in a community setting (i.e., private home or assisted living)	-0.0291	0
Race/Ethnicity		
Non-Hispanic White	-0.2681	0.0005
American Indian/Alaska Native	-0.0043	0.0007
Asian	0.083	-0.0001
Black	0.2092	-0.0002
Native Hawaiian/Pacific Islander	0.073	-0.0008
Hispanic	0.094	-0.0003
Multiracial	0.026	0.0001
**Function and Clinical Characteristics**		
Admit mobility function score (0-15)	-0.0584	-0.0003
Admit self-care function score (0-23)	-0.0844	0
Cognitive Impairment, N (%)		
None	0.1072	-0.0011
Mild	-0.0285	0.0002
Moderate to Severe	-0.092	0.001
Weighted Elixhauser Comorbidity Index (-19 to 89)	-0.0086	0.0006
Pain that interferes with activity or movement	-0.0438	-0.0016
Pressure ulcer at HH admit	-0.0223	0.002
Surgical wound at HH admit	0.0236	-0.0005
Dyspnea Level	-0.0436	-0.0009
Incontinence level		
Not incontinent	0.0851	-0.0001
Incontinent	-0.0816	-0.0002
Requires a urinary catheter	-0.0102	0.0009
History of 2+ falls or injurious fall in last 12 months	-0.0296	-0.0001
History of 2 or more hospitalizations in the past 6 months	-0.0039	0.0001
History of 2 or more ED visits in the past 6 months	-0.003	0.0004

© 2024 Prusynski RA et al. *JAMA Health Forum.*

Currently taking 5 or more medications	-0.0266	-0.001
Cognitive or Behavioral Symptoms Occurring at least once/week	-0.0997	0.0016
**Home and Community Environment**		
Availability of Assistance at Home		
None	0.0263	-0.0007
Some	0.0917	-0.0004
Around the Clock	-0.0976	0.0006
Lives Alone	0.0714	-0.0008
Social Deprivation Index (1-100), Mean (SD)	0.2484	-0.0012
Rurality		
Urban	-0.0281	0.0015
Large Rural	0.0496	-0.0002
Small Rural	-0.0133	-0.0007
Isolated Rural	-0.0248	-0.0031

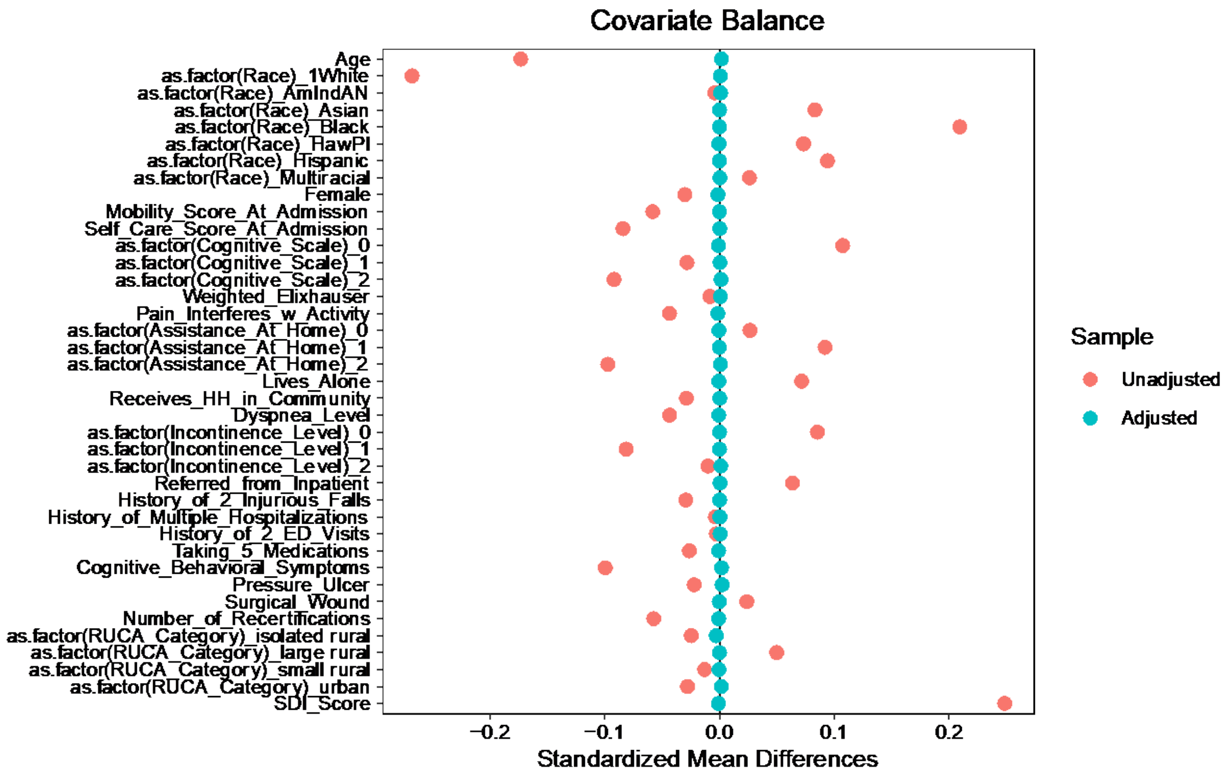

Appendix Figure 1. Standardized mean differences of covariates between Medicare Advantage and Traditional Medicare patients before and after adjustment with inverse probability of treatment weights.

© 2024 Prusynski RA et al. *JAMA Health Forum.*

Appendix Table 2. Estimated adjusted differences in probabilities of outcomes for home health Medicare Advantage (MA) beneficiaries compared to Traditional Medicare

**Outcome**	Coefficient	95% CI	p-value
Improvement in Mobility Function	-0.18%	-0.002%, -0.36%	0.048
Improvement in Self-Care Function	-0.23%	-0.04%, -0.4%	0.017
Discharge to Community	0.29%	0.09%, 0.50%	0.005
Transfer to Inpatient Facility During Episode	0.09%	-0.12%, 0.31%	0.41

Linear probability models include inverse probability of treatment weights that include all patient, social, and environmental characteristics from Table 1 as well as branch and year fixed effects, an indicator for whether the home health episode spanned the implementation of the Medicare Patient-Driven Groupings Model in January 2020, and robust standard errors

© 2024 Prusynski RA et al. *JAMA Health Forum.*
