# Supplementary material for: Differences in Home Health Services and Outcomes Between Traditional Medicare and Medicare Advantage
Source: JAMA Health Forum. 2024 Mar 1;5(3):e235454. doi: 10.1001/jamahealthforum.2023.5454 (PMC10907922; doi:10.1001/jamahealthforum.2023.5454)
Supplement: Supplement 2. — Data sharing statement [file jamahealthforum-e235454-s002.pdf]

Data Sharing Statement

Prusynski. Differences in Home Health Services and Outcomes Between Traditional Medicare and Medicare Advantage. *JAMA Health Forum*. Published March 01, 2024. doi:10.1001/jamahealthforum.2023.5454

Data
Data available: No

Additional Information
Explanation for why data not available: Data were de-identified and provided via a data use agreement with BAYADA Home Health
